# Evaluation of cross-linked aggregates from purified *Bacillus subtilis *levansucrase mutants for transfructosylation reactions

**DOI:** 10.1186/1472-6750-9-68

**Published:** 2009-07-27

**Authors:** Maria Elena Ortiz-Soto, Enrique Rudiño-Piñera, Maria Elena Rodriguez-Alegria, Agustin Lopez Munguia

**Affiliations:** 1 Departamento de Ingeniería Celular y Biocatálisis, Instituto de Biotecnología, UNAM, Cuernavaca, Morelos, 62210, México; 2 Departamento de Medicina Molecular y Bioprocesos, Instituto de Biotecnología, UNAM, Cuernavaca, Morelos, 62210, México

## Abstract

**Background:**

Increasing attention has been focused on inulin and levan-type oligosaccharides, including fructosyl-xylosides and other fructosides due to their nutraceutical properties. *Bacillus subtilis *levansucrase (LS) catalyzes the synthesis of levan from sucrose, but it may also transfer the fructosyl moiety from sucrose to acceptor molecules included in the reaction medium. To study transfructosylation reactions with highly active and robust derivatives, cross-linked enzyme aggregates (CLEAs) were prepared from wild LS and two mutants. CLEAs combine the catalytic features of pure protein preparations in terms of specific activity with the mechanical behavior of industrial biocatalysts.

**Results:**

Two types of procedures were used for the preparation of biocatalysts from purified wild type LS (WT LS) *B. subtilis *and the R360K and Y429N LS mutants: purified enzymes aggregated with glutaraldehyde (cross-linked enzyme aggregates: CLEAs), and covalently immobilized enzymes in Eupergit C^®^. The biocatalysts were characterized and used for fructoside synthesis using xylose as an acceptor model. CLEAs were able to catalyze the synthesis of fructosides as efficiently as soluble enzymes. The specific activity of CLEAs prepared from wild type LS (44.9 U/mg of CLEA), R360K (56.5 U/mg of CLEA) and Y429N (1.2 U/mg of CLEA) mutants were approximately 70, 40 and 200-fold higher, respectively, than equivalent Eupergit C^® ^immobilized enzyme preparations (U/mg of Eupergit), where units refer to global LS activity. In contrast, the specific activity of the free enzymes was 160, 171.2 and 1.5 U/mg of protein, respectively. Moreover, all CLEAs had higher thermal stability than corresponding soluble enzymes. In the long term, the operational stability was affected by levan synthesis.

**Conclusion:**

This is the first report of cross-linked transglycosidases aggregates. CLEAs prepared from purified LS and mutants have the highest specific activity for immobilized fructosyltransferases (FTFs) reported in the literature. CLEAs from R360K and Y429N LS mutants were particularly suitable for fructosyl-xyloside synthesis as the absence of levan synthesis decreases diffusion limitation and increases operational stability.

## Background

*Bacillus subtilis *LS is a fructosyltransferase (FTF) synthesizing levan, a high molecular weight fructose polymer, by using sucrose as a donor and acceptor of the transferred fructosyl moiety (transfructosylation reaction). In general FTFs can also transfer the fructose residue either to water (hydrolysis reaction) or to an acceptor molecule other than the sucrose or fructan present in the reaction medium (acceptor reaction) [1]. Several applications have been developed for levan, such as an anti-inflammatory agent against skin irritation and as a cell-proliferative agent [2]. However, much attention has been focused on inulin and levan-type oligosaccharides, including fructosides containing xylose, galactose, glucose and mannose due their prebiotic properties [3,4] as well as their potential applications as alternative low caloric sweeteners [5]. An additional application of FTFs is derived from its potential to fructosylate a variety of molecules of industrial interest, particularly for food and pharmaceutical applications [6-8]. Nevertheless, considering its broad reaction specificity, *Bacillus subtilis *LS mutants with reduced levan synthesis activity are required to avoid polymer synthesis and to increase fructosides.

One of the greatest challenges in industrial biocatalyst development is the design of highly active and robust derivatives that are stable over a broad range of operational pH and temperatures, a requirement that is strongly associated with the immobilization method [9]. Carrier-bound enzymes traditionally have been the first option for biocatalyst development, even when the inert supports often account for the majority of the biocatalyst mass. In such cases, the concomitant reduction in specific activity results in low volumetric and space-time yields when applied in continuous reactors [10]. A major advancement in biocatalysis is the development of cross-linked enzyme crystals and aggregates (CLECs and CLEAs) that act as biocatalysts that combine the features of pure protein preparations in terms of specific activity and the behavior of biocatalyst particles [9,11-14]. CLECs and CLEAs have been shown to be useful for the development of biocatalysts based on a wide variety of enzymes, including glucose isomerase (D-xylose ketol-isomerase, E.C.5.3.1.5) [15], hydrolases, lyases and oxidoreductases [9,12]. Although CLECs generation requires the crystallization of the enzyme prior to cross-linking, CLEAs preparation from preformed aggregates of purified enzymes constitutes a simpler method with specific activities comparable to CLECs [13]. Moreover, they are particularly advantageous over CLECs in which substrate or products may induce internal diffusional resistances that limit the reaction rate. LS is an interesting option to investigate the design of biocatalysts in the form of CLEAs due to their feasibility for purification as well as their potential for the fructosylation of bioactive compounds.

In this work, we explore xylose fructosylation as a model of acceptor reaction using CLEAs produced from LSs. For this purpose, purified WT LS, R360K and Y429N LS mutants were aggregated and cross-linked. These enzymes were also subjected to traditional immobilization onto Eupergit C^® ^in order to compare the properties and efficiency of both biocatalysts. The properties of WT LS as well as the two mutants have been previously reported [16,17] and are summarized in Table 1. We previously demonstrated that the synthesis of levan displayed by WT LS and R360K (a variant bearing a similar specific activity) can be considerably reduced in the presence of high affinity acceptors such as xylose and maltose, which also reduces their hydrolytic activity [17]. Additionally, Y429N has been reported to be a non-levan-synthesizing mutant that exhibits a 50% reduction in its sucrose hydrolytic activity in acceptor reactions. Therefore, the appropriate enzyme and reaction conditions can be defined for fructosylation reactions with reduced polymer synthesis as well as sucrose hydrolysis, minimizing diffusional limitations by levan.

**Table 1:** Specific activity and reaction specificity of *B. subtilis *WT LS and selected mutants

		**Reaction specificity(%): sucrose (120 g/L)**		**Reaction specificity (%): sucrose (120 g/L)/xylose (120 g/L)**			
**Enzyme**	**Specific activity** **(U/mg protein)**	**Hydrolysis**	**Trans-fructosylation**	**Hydrolysis**	**Trans-fructosylation to sucrose**	**Trans-fructosylation to xylose**	**Product synthesized from sucrose (120 g/L)**

WT LS	160.5 (± 6.0)	52.	547.5	21.3	18.1	60.6	Levan with molecular weight bimodal distribution
R360K	171.2 (± 7.0)	88.3	11.7	37.0	5.3	57.7	Oligosaccharides and low amounts of high molecular weight levan
Y429N	1.5 (± 0.9)	97.3	2.7	49.3	9.6	41.1	Oligosaccharides
Invertase	878.3 (± 6.8)	98.6	1.4	0.0	0.0	0.0	-

^a ^Reaction specificity: percentage of sucrose used for hydrolysis and transfructosylation to sucrose or to xylose. Hydrolysis, transfructosylation and products were determined after 80% sucrose conversion. Data extracted from [17], except for invertase, analyzed in this work.

## Results and Discussion

### CLEAs preparation

**Figure 1:**
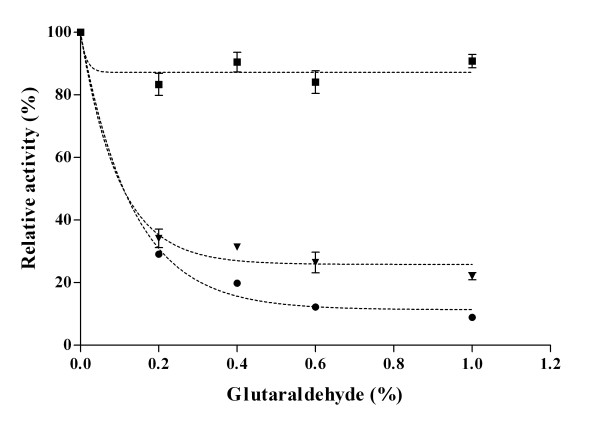
**Effect of glutaraldehyde concentration on relative CLEAs activity**. WT-CLEAs (black circles), R360K-CLEAs (black inverted triangles) and Y429N-CLEAs (black squares). The effect of cross-linking was examined in the range of 0.2 to 1% glutaraldehyde (v/v), using 1 mg/mL of purified enzymes. Activity after cross-linking was compared to the activity of soluble enzymes, which was taken as 100%. Results represent the mean (± SD) of five experiments.

CLEAs were designed to obtain a constrained but sufficiently large levansucrase arrangement to allow for substrate and product diffusion. For this purpose the effect of ammonium sulfate, glutaraldehyde and protein concentrations on CLEAs activity was explored. After solvent accessibility analysis using the ASAView algorithm, it was found that *B. subtilis *LS contains 40 lysine residues with their ε-amino groups exposed to the solvent, including K114, 185 and 363, which are positioned at the rim of the active site. It is therefore important to define proper reaction conditions with glutaraldehyde to avoid excessive cross-linking, as glutaraldehyde mainly reacts with ε-amino groups followed by α-amino, guanidinyl, secondary amino, and hydroxyl groups at near neutral pH [18]. As shown in Figure 1, the highest activity was obtained in all LS-CLEAs when a protein:glutaraldehyde molar ratio of 1:263 (0.2% v/v glutaraldehyde) was used. An important decrease in activity as a result of aggregation was observed for WT LS and R360K CLEAs at glutaraldehyde concentrations higher than 0.2% (v/v). Interestingly, high glutaraldehyde concentrations did not have any effect on Y429N-CLEAs activity, and the immobilized enzyme retained around 90% of soluble enzyme activity. This mutant has a low specific rate and sucrose affinity (kcat and Km of 6 s^-1 ^and 320 mM, respectively, compared to 165 s^-1 ^and 8 mM of the WT LS [17]). These results suggest that WT LS- and R360K-CLEAs undergo diffusional limitations. This could be overcome either by reducing the aggregate particle size, as reported for trypsin-CLEAs [19], where 90% of free trypsin activity was recovered after decreasing the biocatalyst size, or by reducing the amount of enzyme used in the design of the CLEAs. As far as specific activity is concerned, the highest value was obtained with R360K-CLEAs, followed by WT and Y429N-CLEAs, when CLEAs were prepared with 60% ammonium sulfate, 0.2% glutaraldehyde and 4 mg protein/mL. These results are shown in Table 2, where it may be observed that even in the cases of low immobilization efficiency the specific activity of the LSs-CLEAs were the highest reported for an immobilized transglycosylase, either glucosyltransferase (GTF) or FTF, due to the nature of the biocatalyst structure (the proportion of glutaraldehyde in the purified enzymes is negligible) [20,21].

**Table 2:** Characterization of Levansucrases-CLEAs

**Enzyme**	**Aggregation yield^a ^(%)**	**LS-CLEAs** **U/mg of CLEA**
WT LS	28 (± 4.2)	44.9 (± 6.9)

R360K	33 (± 4.1)	56.5 (± 7.2)

Y429N	80 (± 7.5)	1.2 (± 0.1)

^a ^Aggregation yield is defined as the activity recovered in CLEAs when compared with the total activity load of soluble enzyme in the experiment (100%).

**Figure 2:**
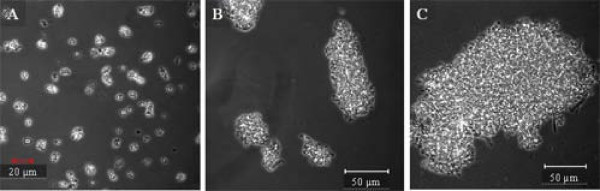
**Images of *B. subtilis *levansucrase R360K mutant CLEAs**. A) CLEAs images obtained immediately after quenching the cross-linking reaction; B) CLEAs washed and centrifuged after cross-linking reaction and C) CLEAs after 48 h of quenching; in this last case, CLEAS were not washed. Digital images were collected in phase contrast mode with a 40×, NA 0.75, Ph 2 objective.

In terms of particle size, the preparation procedure plays an important role, as shown in Figure 2. Indeed, although aggregates have an average size of 15 μm, they tend to form larger aggregates when centrifuged or when residual glutaraldehyde is not washed off.

### Immobilization on Eupergit C^®^

Immobilization of FTFs and GTFs on various supports as Eupergit^®^, hydroxyapatite and alginates has been previously reported for oligosaccharides and polymer synthesis [20-24]. To compare the properties and efficiency of biocatalysts obtained by covalent immobilization on a given support or by aggregation, WT LS and its mutants were also immobilized on Eupergit C^®^. A two-step binding mechanism for the Eupergit C^® ^immobilization process has been proposed. It is assumed that, in the first step, the enzyme is physically adsorbed on the carrier via hydrophobic interactions. This brings the amino and thiol groups on the surface of the enzyme in close proximity to the oxirane groups of the carrier. In the second step, they react with the oxirane groups through nucleophilic attack [25].

The immobilization conditions, including protein concentration, pH and immobilization time, were varied for the R360K mutant, which displayed the highest specific activity. More protein was loaded at pH 7.0 than at pH 6.0 after 38 h of immobilization. In addition, the highest R360K-Eupergit C^® ^specific activity was found at a protein/support ratio of 20/1 (mg protein/g support). A summary of the immobilization procedure on Eupergit C^® ^and the results are shown in Table 3. The specific activity of R360K-CLEAs is higher when compared to the covalent biocatalyst prepared in this study or to previously reported transglycosidases biocatalysts immobilized on the same support [20,21]. Therefore, during biocatalyst design using stable and readily purified enzymes, such as *B. subtilis *LS, it is important to consider the remarkable difference between specific activities of the biocatalysts prepared using inert supports and those obtained as CLEAs. In the case of LS, the latter was about 70, 200 and 40-fold higher for WT LS, Y429N and R360K respectively than the corresponding Eupergit C^® ^immobilized enzyme preparations (Tables 2 and 3).

**Table 3:** Characterization of WT, R360K and Y429N LS immobilized on Eupergit C^®^.

**Enzyme**	**mg LS/g Eupergit C^®^**	**Immobilization yield (%)^d^**	**U LSs/mg EupergitC^®^**
WT LS^a^	6.8	59	0.64

Y429N^a^	7.8	55	0.006

R360K^a^	7.1	60	0.74

R360K^b^	9.3	58	0.93

R360K^c^	18.8	51	1.64

^a ^Immobilization at pH 6.0, during 38 h.^b ^Immobilization at pH 7.0, during 38 h. Immobilization at pH 7.0, during 72 h. In all cases 10 mg of enzyme were contacted per g of Eupergit C^®^, except for (c), where 20 mg were used.^d^Yield is defined as the activity recovered in Eupergit C^® ^when compared with the total activity load of free enzyme in the assay (100%). Eupergit is reported in dry basis.

### Transfructosylation reactions with CLEAs

**Figure 3:**
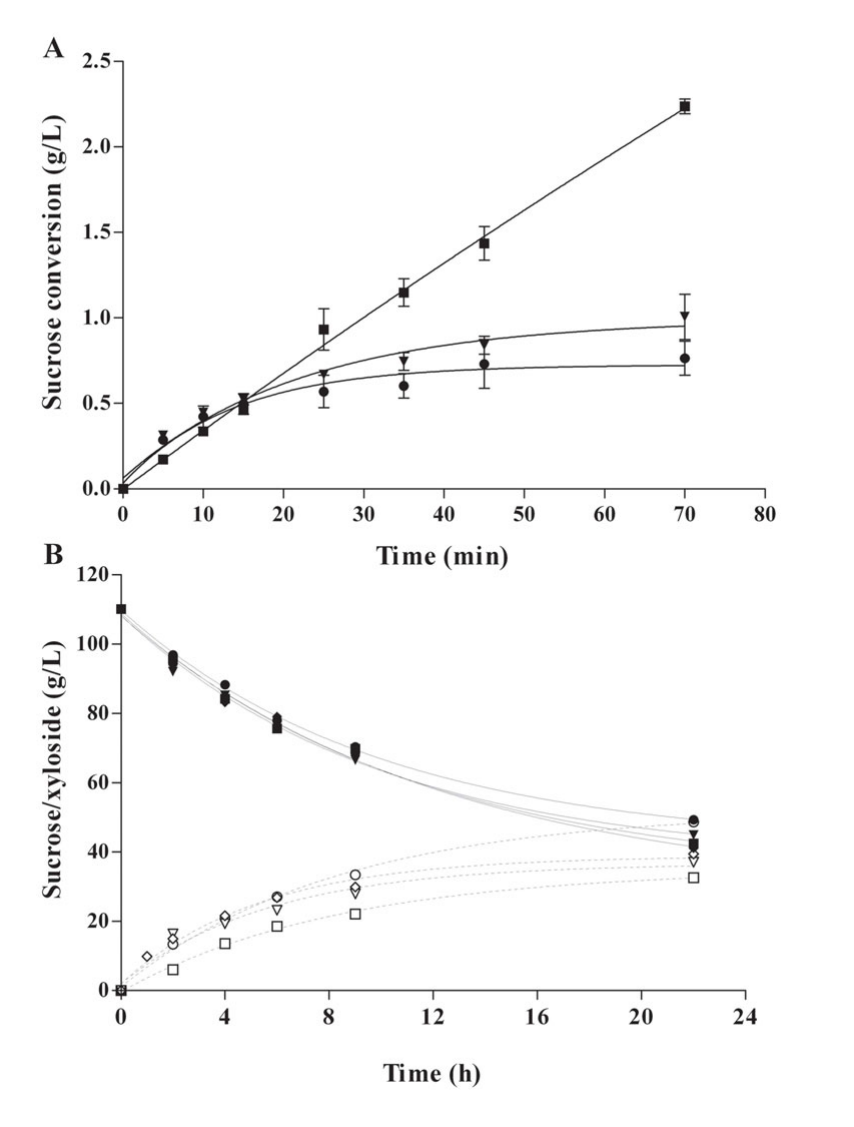
**Reaction evolution during oligosaccharides/levan (A) and fructosyl-xyloside (B) synthesis**. Free R360K (rhombus), WT-CLEAs (circles), R360K-CLEAs (inverted triangles) and Y429N-CLEAs (squares). Black symbols indicate sucrose consumption, while open symbols represent the fructosyl-xyloside synthesis. WT and R360K-CLEAs undergo diffusional restrictions when sucrose is used both as donor and acceptor due to the levan synthesis (A), while, in the presence of xylose, all LS-CLEAs synthesize efficiently the fructosyl-xyloside (B). Reactions for oligosaccharides/levan and fructosyl-xyloside synthesis were carried out with 0.1 and 0.5 U/mL of enzymatic activity respectively. Results represent the mean (± SD) of three experiments.

CLEAs activity was assayed for both levan (or oligosaccharides) and acceptor reactions performed in the presence of sucrose and sucrose/xylose respectively. As expected, WT-CLEAs activity drastically decreases after a few minutes when sucrose acts both as a donor and an acceptor of the fructosyl moiety. It is clear that as the enzyme catalyzes the synthesis of levan, the polymer formed inside the biocatalyst introduces increasing diffusional restrictions as demonstrated by the sudden reduction of sucrose conversion during the reaction (Figure 3A). Although R360K was first reported as a non polymer-producing mutant [16], slight residual polymer-synthesizing activity was retained under our experimental conditions [17]. It is therefore likely that the low amount of high molecular weight levan synthesized by this mutant is enough to introduce internal diffusional resistance in the biocatalyst, particularly when accumulated after a certain operation time or repeated reaction cycles. In contrast, it may also be observed in Figure 3A that sucrose conversion by Y429N, a highly hydrolytic and non-levan synthesizing mutant, continued to increase during the course of the experiment.

It has been reported that D-xylose is a good acceptor and forms β-D-fructofuranosyl-α-D-xylopyranoside when using LS from *B. subtilis *NCIMB 11871 [5]. Additionally, we previously observed that transfer onto sucrose (levan production) is nearly avoided when xylose is added to the reaction medium [17]. We therefore selected xylose as an acceptor model for transfructosylation reactions.

It was also found that all LS-CLEAs catalyzed fructosyl-xyloside production as efficiently as soluble enzymes. Fructosyl-xyloside production from soluble R360K and WT/R360K/Y429N-CLEAs is shown in Figure 3B. Therefore, efficient CLEAs design for fructosylation depends mainly on the selection of adequate LSs and the optimization of reaction conditions to avoid levan synthesis.

**Figure 4:**
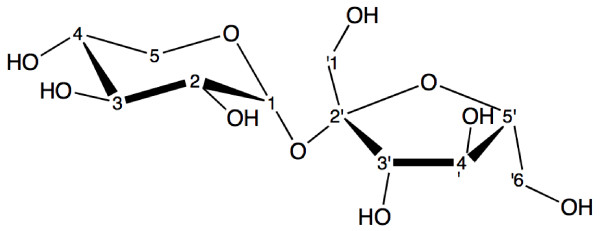
**β-D-fructofuranosyl-(2-1)-α-D-xylopyranoside structure**. The fructosyl-xyloside was purified by HPLC and analyzed by ^1^H and ^13^C NMR spectroscopy, as well as by 2DNMR spectroscopy.

The resulting fructosyl-xyloside was purified, quantified by HPLC and its structure elucidated by ^1^H and ^13^C NMR spectroscopy, as well as by 2DNMR spectroscopy. It was found that *B. subtilis *WT LS forms β-D-fructofuranosyl-(2-1)-α-D-xylopyranoside (Figure 4) when D-xylose acts as acceptor of the sucrose fructosyl moiety, which corresponds to published reports for *B. subtilis *NCIMB 11871 LS [5]. The NMR assignations corresponding to the β-D-fructofuranosyl-(2-1)-α-D-xylopyranoside structure are as follows:

^1^H NMR (400 MHz, D_2_O) 5.3 (*d*, J_1,2 _3.6 Hz, 1H, H-1), 4.2 (*d*, J_3,4_, 8.8 Hz, 1H, H-3'), 4.09 (*t*, J_4,3 _8.4 J_4,5 _8.4 Hz, 1H, H-4'), 3.84–3.88 (*m*, 1H, H-5'), 3.77–3.81 (2d, J_6a, 5 _= J_6b, 5 _2.4 Hz, 2H, 6a-H', 6b-H'), 3.63–3.66 (*m*, 1H, H-4), 3.66–3.69 (m, 4H, H-5, 2H6_a, b_'), 3.57–3.61 (m, 1H, H-3), 3.50 (*dd*, J_2,1 _3.6, J_2,3 _9.6,1H, H-2).

^13 ^C NMR (400 MHz, D_2_O) 105.9 (C-2'), 94.5 (C-1), 83.7 (C-5'), 78.3 (C-3'), 75.3 (C-4'), 75.04 (C-4), 73.3 (C-2), 71.5 (C3), 64.07 (C-5), 64.04 (C-1'), 63.0 (C-6').

### CLEAs' thermal and operational stability

**Figure 5:**
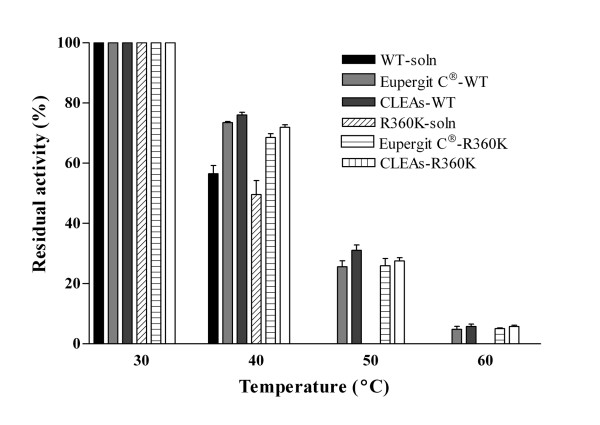
**Residual activity of CLEAs and Eupergit immobilized enzyme preparations of levansucrase and R360K mutant after incubation at several temperatures**. Residual activity was measured after incubation of 2 mg/mL of all preparations at the indicated temperature. Soluble levansucrase is included as a control. Results represent the mean (± SD) of three experiments.

**Figure 6:**
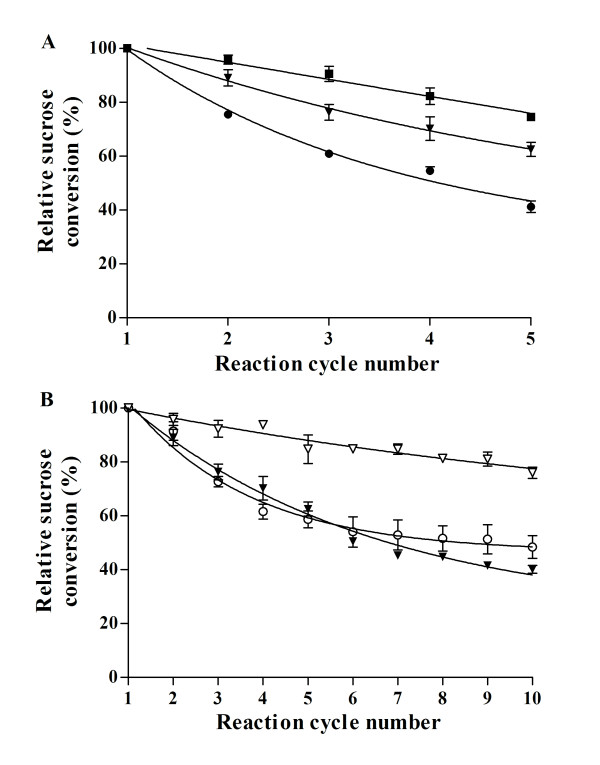
**Operational stability of LSs-CLEAs (A) and Eupergit C^® ^immobilized enzyme preparations (B)**. WT-CLEAs (black circles), WT LS-Eupergit C^®^(open circles), R360K-CLEAs (black inverted triangles), R360K-Eupergit C^® ^(open inverted triangles) and Y429N-CLEAs (black squares). Sucrose conversion in reactions containing 4 U/mL of initial enzymatic activity, 120 g/L of sucrose and 120 g/L of xylose was measured after each 3 h batch-reaction cycle. Sucrose conversion after the first cycle was around 81% (± 7.3) for all CLEAs. R360K-CLEAs are included in both figures. Results represent the mean (± SD) of three experiments.

CLEAs' residual activity after a 1-h incubation at different temperatures in the absence of sucrose was measured with soluble WT LS and the R360K mutant, as well as with the corresponding CLEAs and Eupergit C^®^immobilized enzyme preparations. No significant differences in activity were observed, demonstrating that LS and its R360K mutant display the same thermal stability aggregated in CLEAs than when covalently bound to Eupergit C^® ^(Figure 5). However, when sucrose conversion was measured after consecutive 3-h batch-cycles, it was found that the R360K- and Y429N-CLEAs were more stable than WT LS-CLEAs (Figure 6A).

The apparent activity loss after each reaction cycle is most likely due to product accumulation inside the biocatalyst acting to reduce the global reaction rate and block the intra particle structure of the biocatalysts. Evidence of this phenomenon was found when fructose was identified as a product of acid hydrolysis of deactivated CLEAs (results not shown). Again, Y429N-CLEAs was the more stable biocatalyst, thus complete inhibition of polymer synthesis is required for the maximal biocatalyst operational stability. However, invertase from baker's yeast, which can be considered a fructosidase with little transfructosidase activity is not able to synthesize fructosyl-xyloside (Table 1).

Whereas the activity loss in R360K-CLEAs was about 60% after 10 reaction cycles, Eupergit C^®^-R360K retained about 80%. However, we observed that the activity reduction after each reaction cycle reached a nearly constant sucrose conversion in the last five cycles, indicating that at this time, the residual enzyme is no longer the subject of mass diffusional restrictions and remains stable in subsequent reactions (Figure 6B). For the same reasons, a drastic loss of activity was also observed for WT LS-Eupergit C^®^. Nevertheless, even considering R360K-CLEAs activity loss after 10 reaction cycles, its specific activity is still higher than that of the R360K-Eupergit C^® ^preparation.

## Conclusion

This is the first report of cross-linked transglycosidases aggregates. We have demonstrated that CLEAs are able to catalyze the synthesis of oligofructosides as efficiently as soluble enzymes and display a thermal stability comparable to covalent immobilized enzymes on inert supports. Due to its nature, the LS-CLEAs specific activity reported here is the highest reported for FTFs and GTFs. Moreover, R360K-Eupergit C^® ^specific activity is the highest compared to other FTFs and GTFs immobilized on this support. These biocatalysts are particularly suitable for fructoside synthesis, here demonstrated for β-D-fructofuranosyl-(2-1)-α-D-xylopyranoside production. Reduction of particle size as well as alternative cross-linking conditions should be explored if further reductions in CLEAs diffusional limitations.

## Methods

### Materials

Sugars and invertase (β-fructofuranosidase, EC 3.2.1.26, Grade VII from baker's yeast) were purchased from Sigma Chemical (St. Louis, MO). The electrophoresis reagents were from Bio-Rad Laboratories (CA). All buffer salts were from JT Baker Co. (Phillipsburg, NJ, USA). Eupergit C^® ^was purchased from Röhm Pharma (Darmstadt, Germany) and glutaraldehyde (50%) was from Electron Microscopy Sciences (Fort Washington, PA, USA).

### Bacterial strain, growth conditions and LS purification

*E. coli BL21 *[*E. coli *F^- ^ompT *hsdS*_*B*_(r_B_-m_B_)*gal dcm *(DE3)] transformants were grown, recovered and broken as previously reported [17]. WT and mutants from LS were purified by ion exchange chromatography as previously described [26].

### Protein determination

Protein concentration was determined using the Bradford method (Bio-Rad protein assay) using bovine serum albumin (Albumin fraction V, Sigma Chemical, St. Louis, MO) as the protein standard.

### Activity Assay

Initial reaction rates from free levansucrases, CLEAs and Eupergit C^® ^immobilized enzyme preparations were measured by following the reducing power released from 120 g/L of sucrose in pH 6.0, 50 mM sodium phosphate buffer at 37°C, using the 3,5-dinitro-salicylic acid method (DNS). One unit of enzyme activity was defined as the amount of enzyme releasing one μmol of glucose equivalent per min. The measured activity corresponds in all cases to global LS activity, as it includes released reducing sugars both by transfructosylation (glucose) and hydrolysis (fructose and glucose) activities. Hydrolysis and transfructosylation ratios were determined by specific quantification of glucose and fructose by HPLC.

### CLEAs preparation

Cross-linking optimization reactions were carried out after precipitation of the purified proteins with 60% w/v ammonium sulfate, pH 7.0, 0.1 M sodium phosphate buffer for 30 min; the effect of cross-linking with glutaraldehyde was explored in the 0.2 to 1% concentration range in a total volume of 100 μL for a purified enzyme concentration of 1 mg/mL, while 0.2% glutaraldehyde was used when the protein concentration was varied from 1 to 6 mg protein/mL for 30 minutes at 15°C; after cross-liking the mixture was quenched with 900 μL of 50 mM sodium phosphate buffer, pH 6.0.

Quenched samples were assayed for enzyme activity. A sample was withdrawn from the resulting suspension, which contains both CLEAs and residual free enzyme, and assayed for activity. Afterwards, another aliquot was withdrawn from the suspension, and the CLEAs were removed via centrifugation to obtain activity only from the free enzyme. The difference in activities between the two samples is the CLEAs activity. This approach is the most accurate way of determining the activity of the CLEAs, without having to wash and redisperse, which will increase clotting and thereby mass-transport limitations [10].

Reactions using sucrose both as a donor and as acceptor were carried out with an enzymatic activity of 0.1 U/mL in a medium containing 120 g/L of sucrose in pH 6.0, 50 mM sodium phosphate at 37°C. CLEAs preparation, as well as the activity assays were performed five times.

### Immobilization of WT LS and R360K and Y429N mutants onto Eupergit C^®^

One gram (dry weight) of Eupergit C^® ^(average particle size distribution 100–200 μm, approximate pore diameter 0.1–2.5 μm and specific surface 10–20 m^2^/g) and 10 or 20 mg of purified enzymes were mixed and shaken for 38 or 72 h respectively at 20°C at pH 6.0 or 7.0, in 1 M sodium phosphate buffer. The Eupergit C^® ^beads were recovered by filtration and washed with pH 6.0, 50 mM sodium phosphate buffer (6 × 10 mL). The recovered immobilized enzyme preparations as well as their supernatants were assayed for activity as previously described. The quantity of unbound protein was determined using the Bradford method.

### Fructosyl-xyloside production

Fructosyl-xyloside synthesis was carried out with an enzyme or biocatalyst concentration adjusted to 0.5 U/mL activity in reactions containing 120 g/L of sucrose and 120 g/L of xylose at pH 6.0 and 37°C. Xylose, fructose, glucose, sucrose and fructosyl-xyloside were quantified by HPLC in a Waters 600E system controller (Waters Corp. Milford, MA) equipped with a refractive index detector (Waters 410), and a Carbohydrate (4.6 × 250 mm) column kept at 35°C, using acetonitrile/water (75:25) as the mobile phase at 1.2 mL/min.

### Biocatalysts stability

Purified proteins or biocatalysts including Eupergit C^® ^immobilized enzyme preparations were incubated (2 mg/mL) at 30, 40, 50 and 60°C in pH 6.0, 50 mM sodium phosphate buffer. Samples were withdrawn after 1 h incubation and assayed for residual activity. Stability assays were performed in triplicate.

### Biocatalysts reutilization

Biocatalyst operational stability (LS-CLEAs and LS-Eupergit C^®^) was studied using sequential 1 mL reactions containing a total enzymatic activity of 4 U/mL of either WT LS, R360K or Y429N mutant, sucrose (120 g/L) and xylose (120 g/L) in 50 mM sodium phosphate buffer, pH 6.0, at 37°C under agitation at 1100 rpm. After 3 h of reaction, the medium was centrifuged, sucrose conversion determined and the biocatalysts resuspended in fresh reaction medium to perform a new reaction. All of the assays were performed in triplicate.

### NMR spectroscopy

The sample was dissolved in D_2_O (99.96%, Cambridge isotopes). The spectra were recorded on a Varian Mercury 400 Mhz spectrometer, with TPS as internal reference. 1H-1H correlated spectroscopy (COSY), total correlation spectroscopy (TOCSY) and heteronuclear multiple quantum correlation spectroscopy (HMQC) was used to assign signals. Two dimensional heteronuclear multiple-bond correlation spectroscopy (HMBC) and two-dimensional Nuclear Overhauser Effect Spectroscopy (NOESY) were used to assign inter-residue linkage.

### Microscopy

Samples were observed with a Zeiss LSM 510 META confocal microscope fitted to a Zeiss Axiovert 200 M. Digital images were collected in phase contrast mode with a 40×, NA 0.75, Ph 2.

## Abbreviations

CLEAs: Cross-linked enzyme aggregates; CLECs: Cross-linked enzyme crystals; DNS: 3,5-dinitro-salicylic acid; FTF: Fructosyltransferase; GTF: Glucosyltransferase; LS: Levansucrase; WT: Wild type.

## Authors' contributions

MEOS carried out all of the experimental work, participated in the experimental design and wrote the manuscript. ERP contributed in the experimental design and the structural characterization of the WT LS and immobilized mutant preparations. MERA contributed to images analysis, fructosyl-xyloside purification and structure determination. ALM conceived and supervised the project, as well as the manuscript design and revision. All authors read and approved the final manuscript.
